# Adverse Drug Reactions among Children with Tuberculosis in Tashkent, Uzbekistan, 2019

**DOI:** 10.3390/ijerph18147574

**Published:** 2021-07-16

**Authors:** Makhliyo Abdusalomova, Olga Denisiuk, Hayk Davtyan, Jamshid Gadoev, Barno Abdusamatova, Nargiza Parpieva, Abduvohid Sodikov

**Affiliations:** 1 Department of Phthisiology and Pulmonology, Tashkent Pediatric Medical Institute, Tashkent 100140, Uzbekistan; 2 Alliance for Public Health, 01601 Kyiv, Ukraine; o.denisiuk@gmail.com; 3 Tuberculosis Research and Prevention Center, Yerevan 0014, Armenia; haykdav@gmail.com; 4 World Health Organization Country Office, Tashkent 100100, Uzbekistan; gadoevj@who.int; 5 Main Department of Protection of Maternity and Childhood, Ministry of Health of Uzbekistan, Tashkent 100011, Uzbekistan; barnoa@mail.ru; 6 The Republican Specialized Scientific and Practical Medical Center of Phtisiology and Pulmonology, Tashkent 100179, Uzbekistan; nargizaparpieva@gmail.com (N.P.); Tashobl-optd@mail.ru (A.S.)

**Keywords:** SORT IT, tuberculosis management, pediatric tuberculosis

## Abstract

The treatment of childhood tuberculosis can be challenging due to the lack of pediatric drug formulations and monitoring of drug-toxicity in routine settings. There are no published studies from Uzbekistan on the adverse drug reactions (ADR) associated with anti-tuberculosis treatment in children. In this study, we aimed to investigate the ADR associated with anti-tuberculosis treatment in children. This was a cohort study using secondary program data of children treated at the city and regional tuberculosis clinics in Tashkent, Uzbekistan. Of the 302 patients evaluated, 135 (44.7%) reported ADR. New tuberculosis was registered in 277 (92%) patients and 262 (87%) had extrapulmonary tuberculosis. Factors associated with ADR included treatment at a regional hospital (adjusted odds ratio, aOR = 1.75; *p* = 0.026), female sex (aOR = 2.2; *p* = 0.004), and treatment with second-line drugs (aOR = 8.82; *p* < 0.001). The most common ADRs were gastrointestinal disorders (28.5%) followed by hepatitis (8.9%) and dermatologic reactions (8.6%). Most of the ADRs were mild (55.6%) or moderate (43.7%), only one child had severe ADR. Patients with the identified risk factors should be closely monitored during the treatment. We also recommend expansion of ADR surveillance throughout the country for more representative data in the future.

## 1. Introduction

Tuberculosis (TB) is a disease caused by *Mycobacterium tuberculosis* (*MTB*) and is one of the top 10 causes of death worldwide [1]. In 2019, 10 million people developed TB including 1.2 million TB patients in children (<15 years of age). There were 1.4 million deaths due to TB in the same year and 16% of them occurred in children [1]. 

Compared to adults, children have a higher risk of progressing rapidly to active TB disease after infection, and there are also differences in the immune reactivity and pathophysiology which makes the diagnosis of TB more difficult in children compared to adults [2,3].

The main principles of pediatric TB treatment are similar to the treatment of TB in adults [4]. Although the risk of serious adverse drug reactions (ADR) caused by anti-TB drugs is less frequent in children [5], the treatment of childhood TB can still be challenging due to the lack of pediatric drug formulations and the difficulties involved in monitoring toxicity in routine settings [6].

Most of the TB patients in children are present in low-income and lower middle-income countries with limited recourses and the systems for reporting treatment-related ADR are not well established. 

The Republic of Uzbekistan, situated in Central Asia, is among the 18 high-priority countries for TB control in the World Health Organization (WHO) European Region and one of the 30 high burden countries for multidrug-resistant TB (MDR-TB) globally. In 2019, about 2060 multidrug-/ rifampicin- resistant TB (MDR/RR-TB) patients were registered in the country [1]. Amongst the 16,272 notified incident TB patients (new and relapse), 2190 (13.5%) were children aged 0–14 years. The treatment success percentage was 92% among new and relapse patients with first-line TB drugs in 2018. There are no data for treatment outcomes specifically for children in Uzbekistan. For the WHO European Region, TB treatment success among children was 89.3% [1]. Treatment success is influenced by various factors including ADR and their management, which plays a crucial role in ensuring treatment compliance and retention in care. WHO defines an ADR as “any response to a drug which is noxious and unintended, and which occurs at doses normally used in man for prophylaxis, diagnosis, or treatment of disease, or for the modification of physiological function” [7]. ADR increases morbidity as well as the overall cost of care [8].

Therefore, it is important to have a clear understanding of the frequency and severity of ADR as these may influence TB treatment duration, adherence to drugs and if treatment is interrupted or stopped, individual mortality and morbidity or spread of infection in the community [9].

A literature search revealed no published studies from Uzbekistan about ADRs among children treated for TB. This information may be beneficial for improving the current programs for pediatric TB care and treatment in Uzbekistan.

For all HIV-negative children undergoing inpatient treatment in Tashkent City Children TB Hospital (TCCTH) and the Tashkent Regional Children TB Hospital (TRCTH) for the period of 2019, we aimed to investigate: (a) Socio-demographic and clinical characteristics; (b) frequency and spectrum of ADR and proportion of children having ADR; (c) factors associated with the occurrence of the ADR.

## 2. Materials and Methods

### 2.1. Study Design

This was a cohort study using secondary program data obtained from the Tashkent City Children TB Hospital (TCCTH) and the Tashkent Regional Children TB Hospital (TRCTH) for the period of 2019.

### 2.2. Setting

General setting: The Republic of Uzbekistan is a lower middle-income country located in Central Asia with a population of about 33 million. It consists of 13 administrative regions, one autonomous republic (Karakalpakstan) and Tashkent metropolitan area, the capital city.

Specific setting: TB care and control activities in the country are coordinated by the National TB Program (NTP) of Uzbekistan. TB care is free of charge for all patients throughout the country. National treatment guidelines are in line with WHO guidelines. In the provinces, specialized dispensaries/hospitals and specialized TB units located in primary health care (PHC) facilities provide care to TB patients. Apart from the above mentioned facilities, there is also the Republican Specialized Scientific-Practical Medical Center of Phthisiology and Pulmonology (RSSPMCPP) which is the national TB care facility providing the highest level of care to patients throughout the country.

Inpatient TB care for children: This is provided in “pediatric departments” at the provincial TB hospitals and in RSSPMCPP. Additionally, in the Tashkent region and Tashkent city there are two specialized hospitals for children (TRCTH and TCCTH) providing similar TB care. Anti-TB medications (fixed-dose combinations as well as individual drugs) are available for children throughout the country. Treatment regimens used for the TB treatment in children are the same as for adult patients in terms of used medications and duration of treatment both for drug-susceptible and drug-resistant TB. The dosage of drugs for children is calculated based on weight and adjustments are made during the course of the treatment if necessary depending on weight change and/or ADR. In clinically diagnosed patients (for example, using a tuberculin skin test, a Diaskin test or a chest radiograph) where there is no laboratory confirmation of TB, the treatment regimen is selected based on the drug-susceptibility profile of the index TB patient with whom the child is in contact with. 

ADR surveillance: A newly introduced order (2018) on pharmacovigilance has established a system for recording and reporting ADR, serious ADR (SADR), and ADR of special interest (ADRSI). All the treatment facilities are obliged to record ADR and report all SADR and ADRSI to the pharmacovigilance center at the national level.

Details on the patients’ selection criteria for inpatient and outpatient treatment, recommended pediatric TB diagnostic algorithm, recommended pediatric TB treatment algorithms, recommended dosages for pediatric TB drugs formulations, and severity of ADR are presented in Table 1, Table 2, Table 3, Table 4 and Table 5.

### 2.3. Study Population and Period

All HIV-negative children (0–18 years) with TB treated at the Tashkent City Children TB Hospital and the Tashkent Regional Children TB Hospital during 2019, were included in the study. 

### 2.4. Data Collection, Sources, and Statistical Analysis 

The following data was collected from patient cards and forms: Age, sex, history of TB treatment, disease localization, bacteriological confirmation, treatment regimen, facility, area of residence, Mantoux and Diaskin test results, vaccination with BCG, number of documented ADRs, type of most severe ADRs, and grade of severity of ADR. EpiData Entry (version 4.6.0.0, Odense, Denmark) was used for data capture. The data was collected during the period from November 2019 to March 2020. After extraction, data were checked for errors. Inconsistencies detected were resolved by retrieving the source documents.

### 2.5. Analysis and Statistics 

Sociodemographic and clinical characteristics of the study population were summarized with frequencies and proportions. The outcome of interest was any ADR, while age, sex, category, localization of disease, bacteriological confirmation, treatment regimen, comorbidities, facility, area of residence, Mantoux, Diaskin test, and BCG vaccination were considered as potential risk factors. Odds ratios (OR) were calculated to measure the association between risk factors and ADR and the Chi squared test of association was used for testing of statistical significance of deviation of OR from one. Variables with a *p*-value of <0.1 in the unadjusted analysis were chosen for the multivariable regression model. Age and sex were treated as *a priori* risk factors. Adjusted ORs, confidence intervals, and *p*-values were calculated from the final multivariable logistic regression model. All statistical tests were two-tailed and a *p*-value of <0.05 was considered statistically significant. Statistical analysis was completed using the Stata software version 15 (Stata Corp., College Station, TX, USA).

## 3. Results

### Socio-Demographic, Clinical Characteristics, Frequency, and Predictors of Adverse Drug Reactions

Of the 302 children enrolled in the study, 159 (52%) were male. The mean age of participants was 9.9 (4.2) years. Almost half (47%) of all patients were in the age group of 11–17 years. New TB was registered in 277 (92%) patients and most of the patients (262, 87%) had extrapulmonary TB; most of the patients (296, 98%) were clinically diagnosed and 285 (94%) received first-line drugs; 174 (58%) patients had other co-morbidities reported. Among those with documented test results, the Mantoux and Diaskin test were reported positive in 121 (97%) and 170 (92%) patients, respectively. There were 170 (56%) patients enrolled from the Tashkent Regional Children TB Hospital and 132 (44%) from the Tashkent City Children TB Hospital.

Of all the 302 patients enrolled in the study, a total of 135 (44.7%) patients reported having ADR. Females had two times higher odds of development of ADR compared to male patients (aOR = 2.02; *p* = 0.011). As expected, the patients enrolled in the second-line TB treatment had nearly nine times higher odds of ADR, compared to those receiving the first-line TB treatment (aOR = 8.82; *p*= 0.002). Patients who received the treatment at TRCTH had 75% higher odds of ADR than those receiving the treatment at TCCCH (aOR = 1.75; *p* = 0.003) (Table 6).

The Diaskin test result was not included in the final model, as 39% of children were not tested, however, those that tested positive had 14 times increased chance of development of ADR compared to those that tested negative. 

The frequency of ADR is shown in Table 7. A total of 167 (55.3%) patients experienced no ADRs, while 101 (33.4%), 28 (9.3%), and 5 (1.7%) experienced 1, 2 or 3 ADR, respectively. Only one patient experienced five ADR. 

The spectrum of ADR is described in Table 8. The most frequently documented ADR was gastrointestinal disorders accounting for about half of all ADRs, and observed in 28.5% of the patients followed by hepatitis (15.3% of total ADR) and dermatologic reaction (rash/itching) (14.8% of total ADR). 

The severity assessment is described in Table 9. Most of the reported ADRs were of mild severity (55.7%), followed by moderate severity (43.7%), and severe ADR was observed in one patient.

## 4. Discussion

The anti-tuberculosis drugs-induced ADR are less frequent in children than in adults [5]. However, despite the availability of effective treatment regimens which can cure TB, the ADR can prolong hospitalization, lead to discontinuation of treatment, and treatment failure [10]. 

The timely detection of ADR is crucial to manage them adequately and understanding the factors associated with ADR may help develop appropriate ADR prevention and mitigation strategies [11]. 

This is the first study from Uzbekistan reporting ADR in children with TB. We observed that nearly half of the patients developed ADR which is consistent with the data from other studies in adults [12] but is higher compared to the recent study of ADR among children in Pakistan (13.2%) [13]. The ADR incidence in the study from Brazil was 83.5% [14].

Gastrointestinal disorders were the most commonly observed ADRs which accounted for half of all documented ADRs. Our finding is consistent with the results of the study from Pakistan where also gastro-intestinal disorder was the most frequently observed ADRs (65.7%) [13] and somewhat higher compared to the data of ADR in adults from Iran (22%) [15]. Hepatitis was a second most frequently observed ADR and was seen in nearly one in ten patients. Previous studies suggest isoniazid to be associated with hepatitis with a reported incidence of hepatotoxicity ranging from 0.8% to 16.2% patients [13,16,17]. Compared to the study conducted in Pakistan (0.8%) our study showed a higher level of hepatotoxicity [13]. Anti-TB drug-induced hepatotoxicity is a serious ADR which can be deadly if it is not addressed on time. The monthly monitoring of liver function is recommended for patients on anti-TB treatment.

Skin reaction is known to be caused by rifampicin [18]. In our study, dermatological ADR was observed in 8.6% of patients, which was relatively higher than data from Pakistan (0.8%) [13] but lower compared to adults (21%) [19]. Hypersensitivity skin reactions and hepatitis are known to be one of the most difficult ADRs to manage and can lead to the discontinuation of treatment and poor treatment outcomes [20]. 

The study from Pakistan showed that female children, those aged 6–10 years, children with EPTB, previously treated patients, and children without a BCG scar had a higher risk of ADRs [13]. In our study too, female sex was associated with higher odds of ADR [13]. Studies of ADR in adults also showed that the severity of ADR was greater in females [15]. The reasons for this association are unclear. Females tend to have reduced hepatic clearance, a lean body mass, and lower glomerular filtration rate—thus it is not clear if this association occurs due to the differences in pharmacokinetics or due to the other factors. Hence, female patients should be monitored carefully for ADR while on the treatment.

Patients who were treated in the TRCTH hospital were almost twice likely to be registered with ADR as compared to those in the TCCTH. Both clinics are following the same TB treatment and ADR management protocol. We speculate that this might reflect better documentation of ADRs in the patient’s cards. 

Many studies have shown that ADRs are higher in patients who are treated with second-line anti-TB drugs, which is in line with our study finding [21]. This might be due to the toxic nature of the drugs used, longer duration of treatment, and lack of pediatric formulations making it challenging to adjust the dosage based on body weight [22]. 

The Diaskin test indicates the accuracy of the diagnosis and the severity of tuberculosis [23]. Our study shows the association between the positive Diaskin test and the ADR occurrence, which might be due to the severity of the disease. 

Our study has several limitations. First, we had to rely on the program data given the retrospective nature of the study. Hence, we were unable to rule out any errors that may have occurred during data collection or recording. Second, we were not able to collect data on the timing of ADR occurrence and to follow-up on the treatment outcomes and their association with ADR. 

The strength of the study is that we collected and analyzed all the available data for the defined period and it potentially reflects the reality of the pediatric TB care in Uzbekistan. 

## 5. Conclusions

ADRs are frequently seen in children with TB. The medical providers should be attentive in order to prevent, detect ADRs at the earliest, and manage them appropriately. Patients with risk factors associated with ADR, such as females, patients on SLD, and those with a positive Diaskin test should be closely monitored during the treatment. Such surveillance for ADR should be expanded throughout the country so that more representative data can be obtained in the future. All the health care providers involved in pediatric TB care should be educated on ADR detection and management. 

## Figures and Tables

**Table 1:** Patient selection criteria for inpatient and outpatient treatment for children with tuberculosis in Uzbekistan, 2019.

Patient Selection Criteria for Inpatient Treatment	Patient Selection Criteria for Outpatient Treatment
Children (aged 0–17 years) with active tuberculosis in various sites, regardless of bacteriological confirmation, shall undergo inpatient treatment for at least 2 months, while the decision is made on an individual basis when there is cavitary disease with bacterial excretion.	Children (0–17) with milder forms of active tuberculosis, clinically diagnosed patients, non-cavitary disease, and no intoxication symptoms; Children with active forms of tuberculosis without risk factors (cohabitation with a diagnosed TB patient who has a positive sputum smear (SS+), HIV-positive or evident cachexia in a child).

**Table 2:** Recommended pediatric TB diagnostic algorithm for children with tuberculosis in Uzbekistan, 2019.

History	Careful Analysis (Including History of TB Contacts and Symptoms Consistent with TB).
Clinical examination	Assessment of physical development.
Tuberculin immunological skin test	Mantoux test, Interferon-gamma release assays (Diaskin test, TB-Spot test).
Bacteriological	Sputum microscopy, Xpert MTB/RIF assay, line probe assay (HAIN TEST), liquid (MGIT) culture, solid (Löwenstein-Jensen) culture.
X-ray	X-ray, chest fluorography, computer tomography (CT), bone and joints X-rays, magnetic resonance imaging (MRI).
Laboratory testing	Complete blood count, urine test, spinal puncture, fine needle aspiration, test of pleural and ascitic fluid sample, if needed.

**Table 3:** Recommended pediatric TB treatment algorithms for children with tuberculosis in Uzbekistan, 2019.

MTB and Drug Susceptibility Testing	Phases of Chemotherapy Course	
	Intensive Phase	Continuation Phase
MTB+, susceptible to first-line drugs MTB (bacteriologically negative) Strong recommendation	2 HR ZE	4 HR
MTB+, resistant to H or to H in combination with S, Z, E Conditional recommendation	3 RZE Km Lfx	6 RZE Lfx
MDR/RR-TB Strong recommendation	8 Z Km (Cm) Lfx Pto Cs (PAS)	12 Z Lfx Pto Cs (PAS)
Pre-XDR-TB, MDR/RR-TB with resistance to ofloxacin Weak recommendation	6 Bdq 8 Z Km (Cm) Pto Cs PAS	16 Z Pto Cs PAS
Pre-XDR-TB, MDR/RR-TB with resistance to second-line injectables Weak recommendation	8 Z Cm Lfx Pto Cs PAS	16 Z Lfx Pto Cs PAS
XDR-TB Weak recommendation	6 Bdq Cm Lzd PAS Im/Сln Amx/clv	18 Mfx Lzd PAS Amx/clv

MTB: Mycobacterium tuberculosis; H: Isoniazid; R: Rifampicin; Z: Pyrazinamide; E: Ethambutol; S: Streptomycin; Km: Kanamycin; Lfx: Levoflaxacin; MDR-TB: Multidrug-resistant tuberculosis; Cm: Capreomycin; Pto: Protionamide; Cs: Cycloserine; PAS: Para-aminosalicylic acid; XDR-TB: Extensively drug-resistant tuberculosis; Bdq: Bedaquiline; Lzd: Linezolid; Im/Cln: Imipenem with cilastatin; Amx/Clv: Amoxicillin potentiated with clavulanate; Mfx: Moxifloxacin.

**Table 4:** Recommended dosages for pediatric TB drugs formulations for children with tuberculosis in Uzbekistan, 2019.

Drug	Daily Dose (mg/kg)	Frequency	Maximum Daily Dose
Isoniazid	10 (10–15)	Once a day	600 mg 2000 mg
Rifampicin	15 (10–20)	Once a day	600 mg
Pyrazinamide	35 (30–40)	Once a day	2 g
Ethambutol	20 (15–25)	Once a day	1.2–2 g
Streptomycin	20–40	Once a day	1 g
Kanamycin	15–30	Once a day	1 g
Amikacin	15–22.5	Once a day	1 g
Capreomycin	15–30	Once a day	1 g
Levofloxacin	7.5–10	Once a day	750 mg
Moxifloxacin	7.5–10	Once a day	400 mg
Ethionamide	15–20	Twice a day	1 g
Protionamide	15–20	Twice a day	1 g
Cycloserine	10–20	Once or twice a day	1 g
Para-aminosalicylic acid	150	Twice or three times a day	12 g

**Table 5:** Adverse drug reaction severity levels among children with tuberculosis in Uzbekistan, 2019.

Severity Level	Determining the Severity Level
Level 1—mild	Transient or mild discomfort (<48 h); medical intervention/therapy is not required.
Level 2—moderate	Mild to moderate limitations in activity—some assistance is required; medical intervention/therapy is required in minimum or is not required at all.
Level 3—severe	Noticeable limitations in activity, some assistance is usually required; medical intervention/therapy is required, with possible hospitalization.
Level 4—life-threatening	Excessive limitations in activity, extensive assistance is required; extensive medical intervention/therapy is required.

**Table 6:** Socio-demographic, clinical characteristics, and factors associated with the occurrence of the adverse drug reactions among pediatric tuberculosis patients registered at Tashkent City Children Tuberculosis Hospital and Tashkent Regional Children Tuberculosis Hospital, Uzbekistan, 2019.

Characteristics	Total		ADR		No ADR		OR	95% CI	*p*-Value	aOR	95% CI	LRT *p*-Value
	*n*	%	*N*	(%)	*n*	(%)						
Total	302		135	44.7	167	55.3						
Age group												
<5 years	60	(19.9)	23	(38.3)	37	(61.7)	1			1		0.209
5–10 years	101	(33.4)	40	(39.6)	61	(60.4)	1.05	(0.55–2.03)	0.873	1.2	(0.57–2.50)	
11–17 years	141	(46.7)	72	(51.1)	69	(48.9)	1.68	(0.91–3.12)	0.100	1.46	(0.71–3.03	
Sex												
Male	159	(52.6)	60	(37.7)	99	(62.3)	1					0.004
Female	143	(47.4)	75	(52.4)	68	(47.6)	1.82	(1.15–2.88)	0.011	2.02	(1.25–3.27)	
TB category												
New	277	(91.7)	122	(44.0)	155	(56.0)	1					
Retreatment	25	(8.3)	13	(52.0)	12	(48.0)	1.37	(0.61–3.12)	0.445			
TB localization												
Pulmonary	38	(12.7)	18	(47.4)	20	(52.6)	1					
Extrapulmonary	262	(87.3)	117	(44.7)	145	(55.3)	0.9	(0.45–1.77)	0.754			
Missing	2											
Bacteriological confirmation												
Yes	6	(52.6)	2	(33.3)	4	(66.7)	1					
No	296	(47.4)	133	(44.9)	163	(55.1)	1.63	(0.29–9.05)	0.575			
Treatment												
FLD	285	(94.4)	120	(42.1)	165	(57.9)	1		1			<0.001
SLD	17	(5.6)	15	(88.2)	2	(11.8)	10.3	(2.31–45.9)	0.002	8.82	(1.92–40.60)	
Co–morbidities												
Yes	174	(57.6)	79	(45.4)	95	(54.6)	1.07	(0.68–1.69)	0.775			
No	128	(42.4)	56	(43.8)	72	(56.3)	1					
Clinic												
TCCTH	132	(43.7)	46	(34.8)	86	(65.2)	1			1		0.026
TRCTH	170	(56.3)	89	(52.4)	81	(47.6)	2.05	(1.29–3.28)	0.003	1.75	(1.07–2.88)	
Residence												
Urban	140	(46.4)	49	(35.0)	91	(65.0)	1					
Rural	162	(56.3)	86	(53.1)	76	(46.9)	2.1	(1.32–3.34)	0.002			
Had contact with TB patients												
No	153	(51.2)	62	(40.5)	91	(59.5)	1					
Yes	146	(48.8)	71	(48.6)	75	(51.4)	1.19	(0.92–1.55)	0.194			
Missing	3											
Mantoux test												
Negative	4	(3.2)	0	(0.0)	4	(100.0)			0.047			
Positive	121	(96.8)	61	(50.4)	60	(49.6)						
No test done	177											
Diaskin test												
Negative	14	(7.6)	1	(7.1)	12	(85.7)	1					
Positive	170	(92.4)	93	(54.7)	76	(44.7)	14.7	(1.87–115.5)	0.011			
No test done	120											
BCG												
Yes	154	(88.0)	67	(43.5)	87	(56.5)	1.03	(0.81–1.30)	0.803			
No	21	(12.0)	11	(52.4)	10	(47.6)	1					
Missing	127											

ADR: Adverse drugs reaction; OR: Odds ratio; aOR: Adjusted odds ratio; CI: Confidence interval; TB: Tuberculosis; TCCTH: Tashkent City Children Tuberculosis Hospital; TRCTH: Tashkent Regional Children Tuberculosis Hospital; FLD: First-line TB drugs; SLD: Second-line TB drugs; BCG: Bacillus Calmette-Guerin.

**Table 7:** Frequency of adverse drug reactions among pediatric tuberculosis patients registered at Tashkent City Children Tuberculosis Hospital and Tashkent Regional Children Tuberculosis Hospital, Uzbekistan, 2019.

Frequency of ADR	Number	%
No ADR	167	55.3
One ADR	101	33.4
Two ADR	28	9.3
Three ADR	5	1.7
Five ADR	1	0.3
Total	302	100.0

TB: Tuberculosis; ADR: Adverse drug reaction.

**Table 8:** Spectrum of adverse drug reactions among pediatric tuberculosis patients registered at Tashkent City Children Tuberculosis Hospital and Tashkent Regional Children Tuberculosis Hospital, Uzbekistan, 2019.

Types of ADR	Number ADR	% Patients with ADR	% Total ADR
Gastro-intestinal	86	28.5	48.9
Hepatitis	27	8.9	15.3
Dermatological	26	8.6	14.8
Psychiatric, CNS disorder	14	4.6	8.0
Urinary tract disorder	11	3.6	6.3
Bone pain	4	1.3	2.3
Myalgia	3	1.0	1.7
Arthralgia	2	0.7	1.1
Peripheral neuropathy	2	0.7	1.1
Hypokalemia	1	0.3	0.6
Total	176	58.3	100.0

ADR: Adverse drug reaction; CNS: Central nervous system.

**Table 9:** Severity of adverse drug reactions among pediatric tuberculosis patients registered at Tashkent City Children Tuberculosis Hospital and Tashkent Regional Children Tuberculosis Hospital, Uzbekistan, 2019.

Severity	*n*	(%)
Mild	75	55.6
Moderate	59	43.7
Severe	1	0.7

ADR: Adverse drug reaction.

## Data Availability

The data that support the findings of this study are available from the corresponding author, (M.A.), upon reasonable request.

## ©World Health Organization 2021

All rights reserved. The WHO Regional Office for Europe has granted the Publisher permission for the reproduction of this article.

## Open Access Statement

In accordance with WHO’s open-access publication policy for all work funded by WHO or authored/co-authored by WHO staff members, the WHO retains the copyright of this publication through a Creative Commons Attribution IGO license (http://creativecommons.org/licenses/by/3.0/igo/legalcode) which permits unrestricted use, distribution, and reproduction in any medium provided the original work is properly cited.
